# The overexpression and clinical significance of TBX15 in human gliomas

**DOI:** 10.1038/s41598-023-36410-y

**Published:** 2023-06-16

**Authors:** Dongming Yan, Yaping Yu, Qiongwei Ni, Qingwen Meng, Haolin Wu, Shun Ding, Xiaoqian Liu, Caiying Tang, Qibing Liu, Kun Yang

**Affiliations:** 1 Department of Neurosurgery, The First Affiliated Hospital of Hainan Medical University, Haikou, 570102 China; 2 Tumor Institute and Medical Research Central of The First Affiliated Hospital, Haikou, 570102 China; 3 Central South University Xiangya School of Medicine Affiliated Haikou Hospital, Haikou, 570208 China; 4 Department of Cardiovascular Medicine, The First Affiliated Hospital of Hainan Medical University, Haikou, 570102 China; 5 Department of Pharmacology, Hainan Medical University, Haikou, 571199 China; 6 Department of Otolaryngology, Head and Neck Surgery, The First Affiliated Hospital of Hainan Medical University, Haikou, 570102 China; 7 The First Affiliated Hospital Trauma Center, Hainan Medical University, Haikou, 570100 China; 8 Department of Pharmacy, The First Affiliated Hospital of Hainan Medical University, Haikou, 570100 China

**Keywords:** Bioinformatics, Cancer, CNS cancer, Cancer, Biomarkers, Oncology

## Abstract

T-box transcription factor 15 (TBX15) is upregulated in a variety of tumors and has been reported to promote uncontrolled proliferation of tumor cells and induce tumor cells to avoid apoptosis, thus accelerating the malignant transformation of malignant tumors. However, the prognostic value of TBX15 in glioma and its relationship with immune infiltration remain unknown. In this study, we intended to explore the prognostic value of TBX15 and its link to glioma immune infiltration and examine TBX15 expression in pan-cancer using RNAseq data in TPM format from TCGA and GTEx. TBX15 mRNA and protein expressions in glioma cells and adjacent normal tissue were detected and compared by RT-qPCR and Western blot. The effect of TBX15 on survival was assessed by Kaplan-Meier Method. The correlation between TBX15 upregulation and the clinicopathological characteristics of glioma patients was assessed by using TCGA databases, and the relationship between TBX15 and other genes in glioma was evaluated by using TCGA data. The top 300 genes most significantly associated with TBX15 were selected to establish a PPI network through the STRING database. The relationship between TBX15 mRNA expression and immune cell infiltration was explored by using ssGSEA and the TIMER Database. It was found that TBX15 mRNA expression in glioma tissues was significantly higher than that in the adjacent normal tissues, and this difference was most obvious in high-grade gliomas. TBX15 expression was increased in human gliomas and associated with worse clinicopathological characteristics and poorer survival prognosis in glioma patients. In addition, elevated TBX15 expression was linked to a collection of genes involved in immunosuppression. In conclusion, TBX15 played an important role in immune cell infiltration in glioma and may prove to be a predictor of the prognosis in glioma patients.

## Introduction

Glioma is the second leading cause of mortality from central nervous system (CNS) illnesses following stroke. Glioma can affect any part of the CNS, especially the brain and glial tissue^1^. WHO grade 4 glioblastoma (GBM) is the most prevalent and deadly form of glioma, accounting for more than 70% of all brain cancers, with an incidence of 3.2 cases per 100,000 persons^2^. Despite significant advancements in the technical level of surgery, radiation, and chemotherapy for the treatment of GBM during the previous two decades, the prognosis of GBM patients has remained poor. The median survival rate of GBM patients is about 1–1.5 years regardless of therapy, and the five-year survival rate is lower than 5%^3^. New biomarkers may improve the diagnostic accuracy of gliomas, create opportunities for new pathogenesis-based pharmacological treatments and immunotherapy, and then tailor personalized treatments for each tumor characteristic. Therefore, it is critical to investigate the molecular mechanisms underlying glioma's malignant growth and develop therapeutic options for possible targets.

TBX15 belongs to the T-box gene family. The size of T-box proteins is normally between 50 and 78 kDa. These transcription factors are known to regulate a variety of developmental processes, and all genes in this family contain a common T-box DNA binding region. Some downstream target detection and binding site selection tests showed that all T-box proteins were able to bind to the TCACACCT DNA consensus sequence^4^. Recent research has discovered that TBX15 promotes cancer progression in solid tumors such as thyroid cancer, renal clear cell carcinoma, and ovarian cancer^5–9^, but few studies have reported its biological behavior and molecular mechanism in glioma.

**Figure 1:**
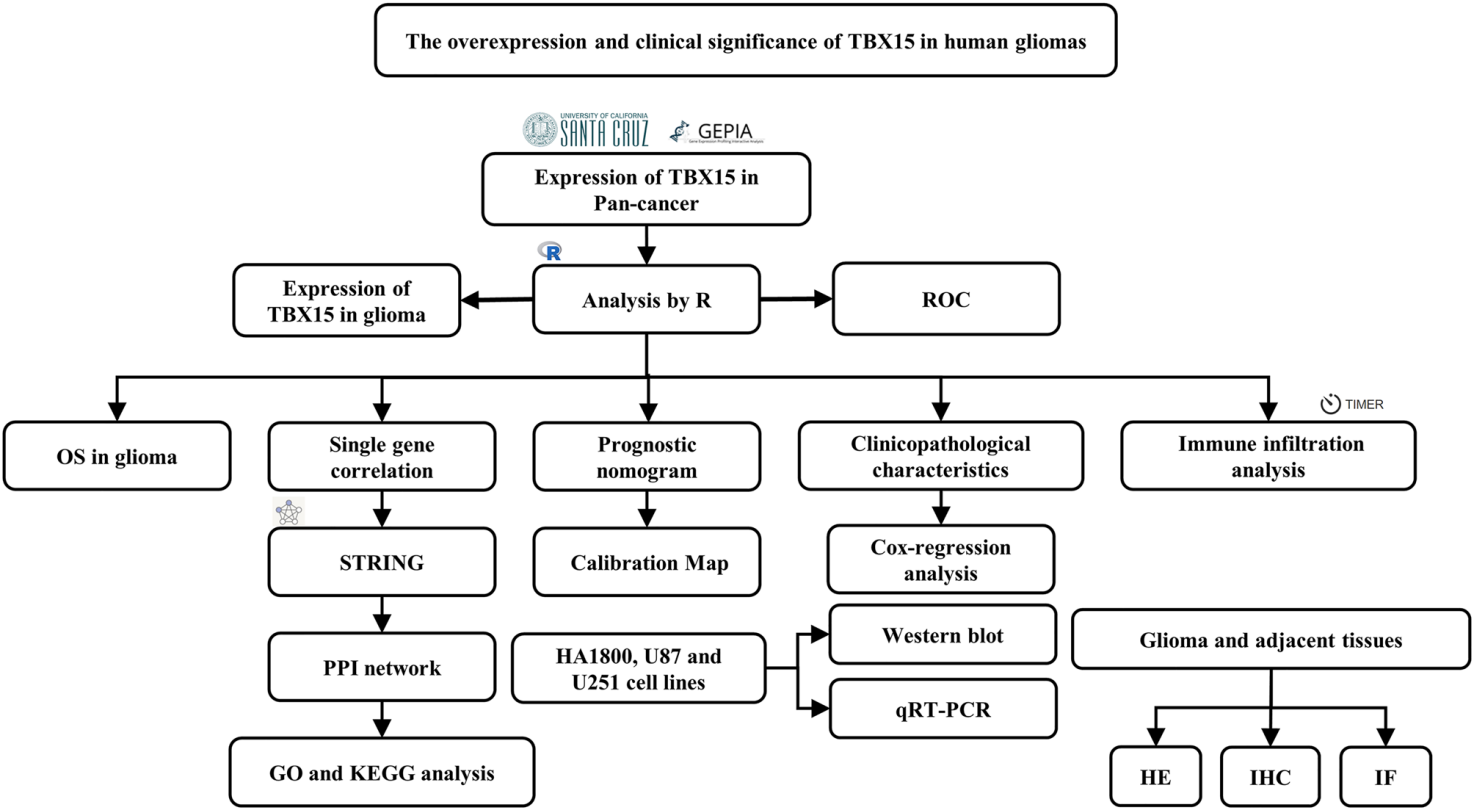
Flowchart of this study.

The present study aimed to evaluate the expression of TBX15 in gliomas in TCGA, and GTEx cohorts, and investigate its potential mechanisms. The process digraph is shown in Fig. 1. It was found that TBX15 expression was remarkably up-regulated in a spectrum of malignancies including glioma, and TBX15 upregulation was associated with poor prognosis of glioma patients. In addition, TBX15b also played a role in extracellular matrix (ECM)-receptor interaction, proteoglycans in cancer, and mesenchymal-epithelial transition (MET) activation of PTK2 signaling, PI3K-Akt signaling pathway, and immune system. Given the importance of immune cell infiltration in the overall survival (OS) of glioma patients, we checked the link between TBX15 overexpression and immune cell infiltration and found that Th2 cell, macrophage, aDC, neutrophil, eosinophil, and T cell infiltrations were all positively correlated with high TBX15 expression in gliomas. Our results demonstrated that TBX15 was highly expressed in gliomas and cell lines, working as an oncogenic protein. These findings suggest that TBX15 overexpression may prove to be a potential prognostic biomarker of glioma patients.

## Results and discussion

### Pan-cancer TBX15 expression analysis

**Figure 2:**
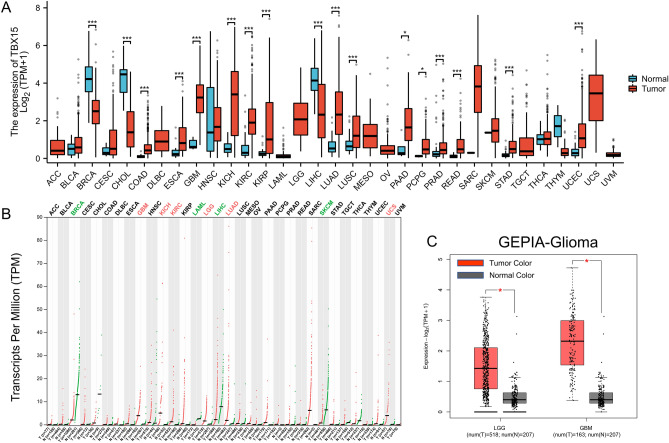
Pan-cancer TBX15 expression analysis. (**A**) TBX15 expression in cancerous and non–cancerous tissues as shown by TCGA and GTEx pan-cancer data. (**B**) GEPIA database Pan-cancer analysis. (**C**) GEPIA database analysis showed that the expression of TBX15 in LGG and GBM tissues was higher than that in normal tissues. (*, *P* < 0.05; **, *P* < 0.01; ***, *P* < 0.001).

We first analyzed the expression of TBX15 mRNA in TCGA and GTEx pan-cancer data, and then compared the results with the GEPIA database and found that TBX15 mRNA was expressed differently in different tumors (Fig. 2A,B). According to the GEPIA database, TBX15 mRNA expression was greater in LGG and GBM tissues than in normal tissues (Fig. 2C).

**Figure 3:**
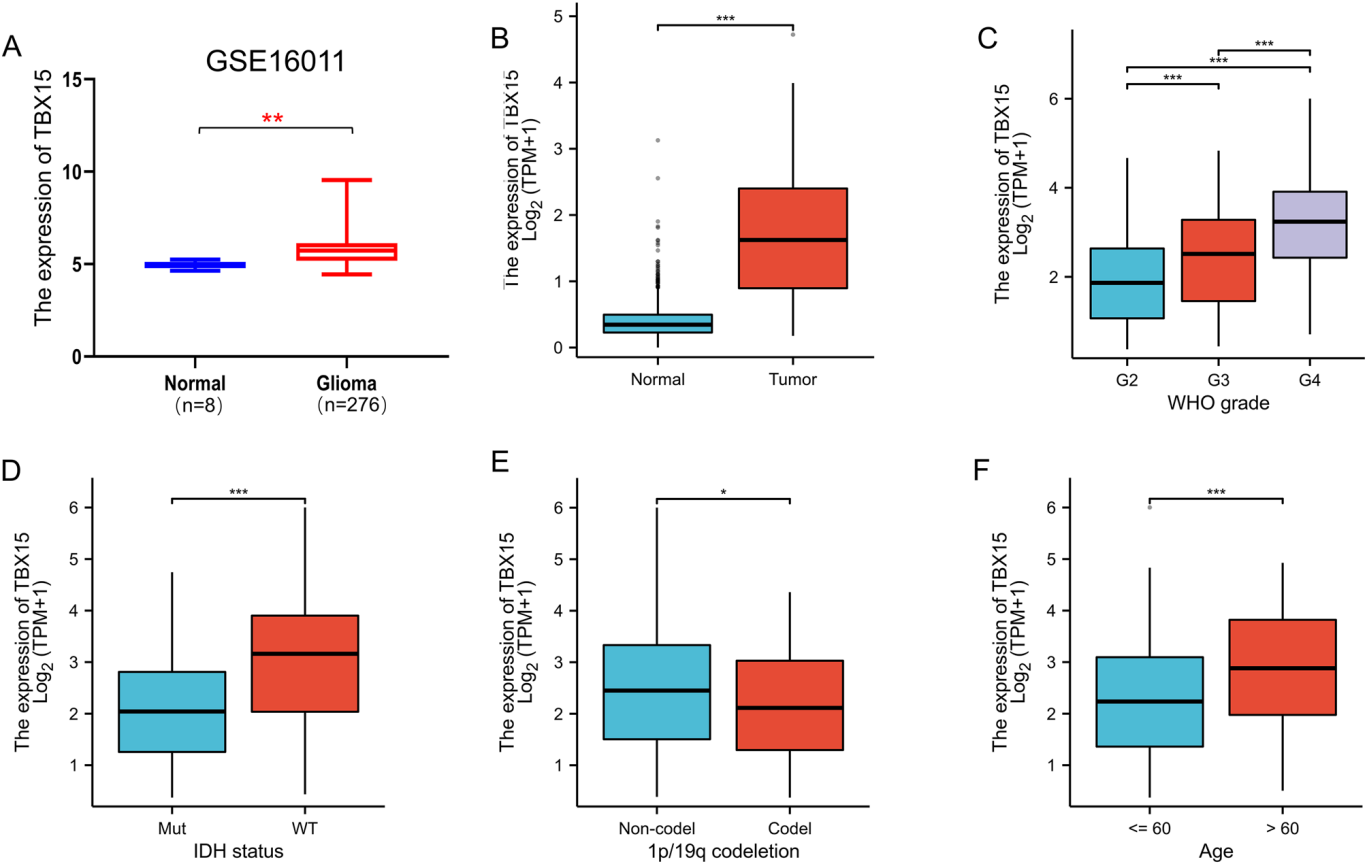
TBX15 mRNA is upregulated in gliomas and is associated with clinicopathological characteristics. (**A**) TBX15 mRNA was remarkably expressed in GSE16011(Platform, GPL8542) glioma. (**B**) TBX15 mRNA was remarkably expressed in TCGA glioma. (**C**–**F**) TBX15 mRNA is upregulated in gliomas and is associated with clinicopathological characteristics. (*, *P* < 0.05; **, *P* < 0.01; ***, *P* < 0.001).

In addition, TBX15 mRNA was remarkably expressed in GSE16011(Platform, GPL8542) glioma (Fig. 3A), this is consistent with the results of our study (Fig. 3B). The expression level of TBX15 mRNA increased with the increase of WHO grade (Fig. 3C), IDH wild type had higher TBX15 mRNA expression than mutant type (Fig. 3D), expression in 1p/19q non-codel was higher than that in codel (Fig. 3E), older people express more than younger people (Fig. 3F). These results suggest that TBX15 mRNA was expressed differently in different tumors, and TBX15 mRNA is upregulated in gliomas and is associated with clinicopathological characteristics.

### ***TBX15 expression is related to the prognosis of glioma patients***

**Figure 4:**
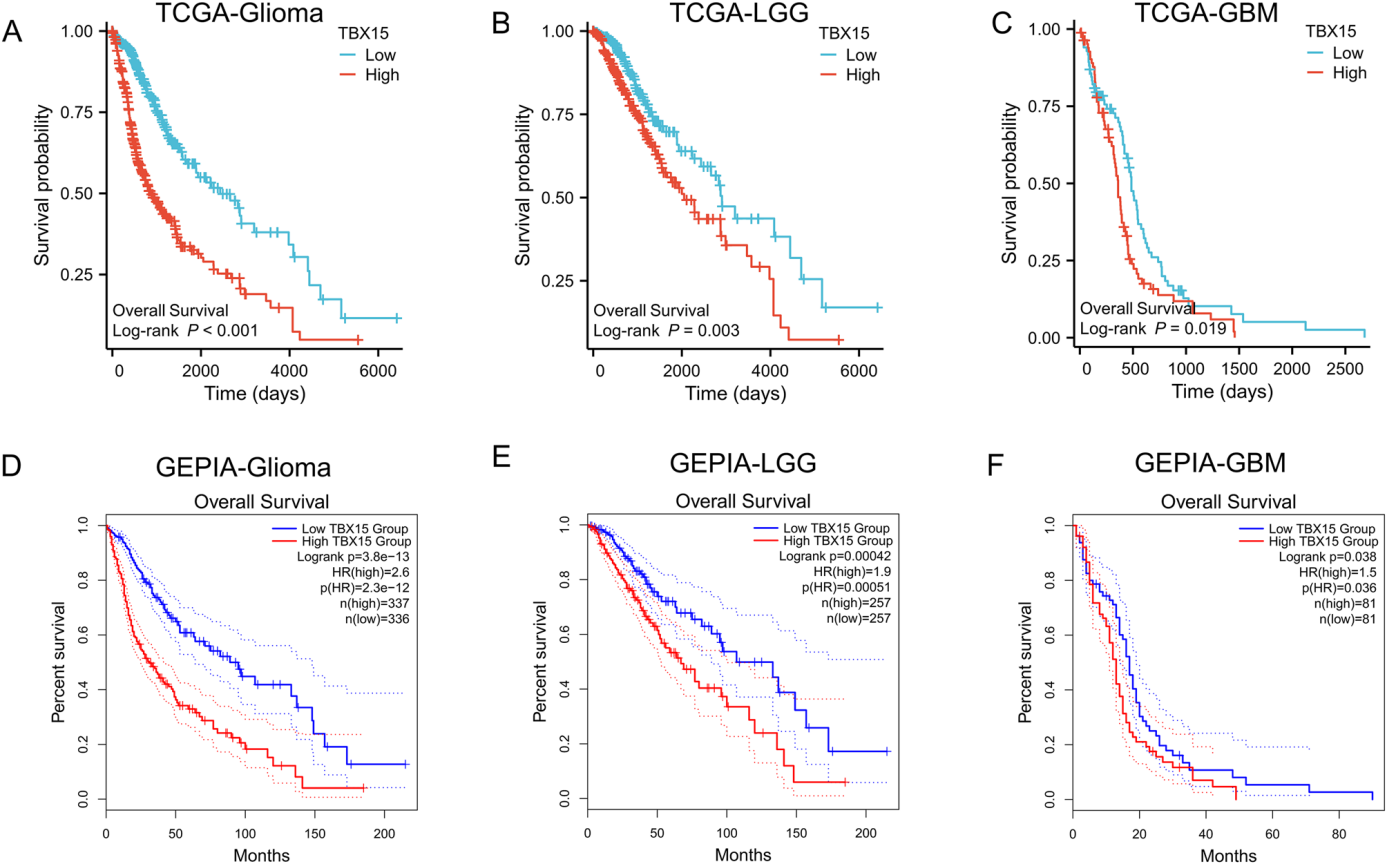
TBX15 expression is related to the prognosis of glioma patients. (**A**–**C**) The Kaplan–Meier method was used to examine OS in the TCGA-glioma. (**D**–**F**) GEPIA database analysis showed that the overall survival in glioma patients in the TBX15 mRNA high expression group was significantly poorer than that in those of the TBX15 mRNA low expression group.

The link between TBX15 expression and OS was examined in the TCGA-glioma cohort to determine the utility of TBX15 in predicting glioma patient prognosis. A higher TBX15 mRNA expression was related to a poorer OS in glioma (Fig. 4A–C). We get the same result based on the GEPIA database (Fig. 4D–F). These results suggest that TBX15 mRNA can be used as a marker to evaluate the prognosis of glioma.

### TBX15 expression is increased in glioma cells and tissues

**Figure 5:**
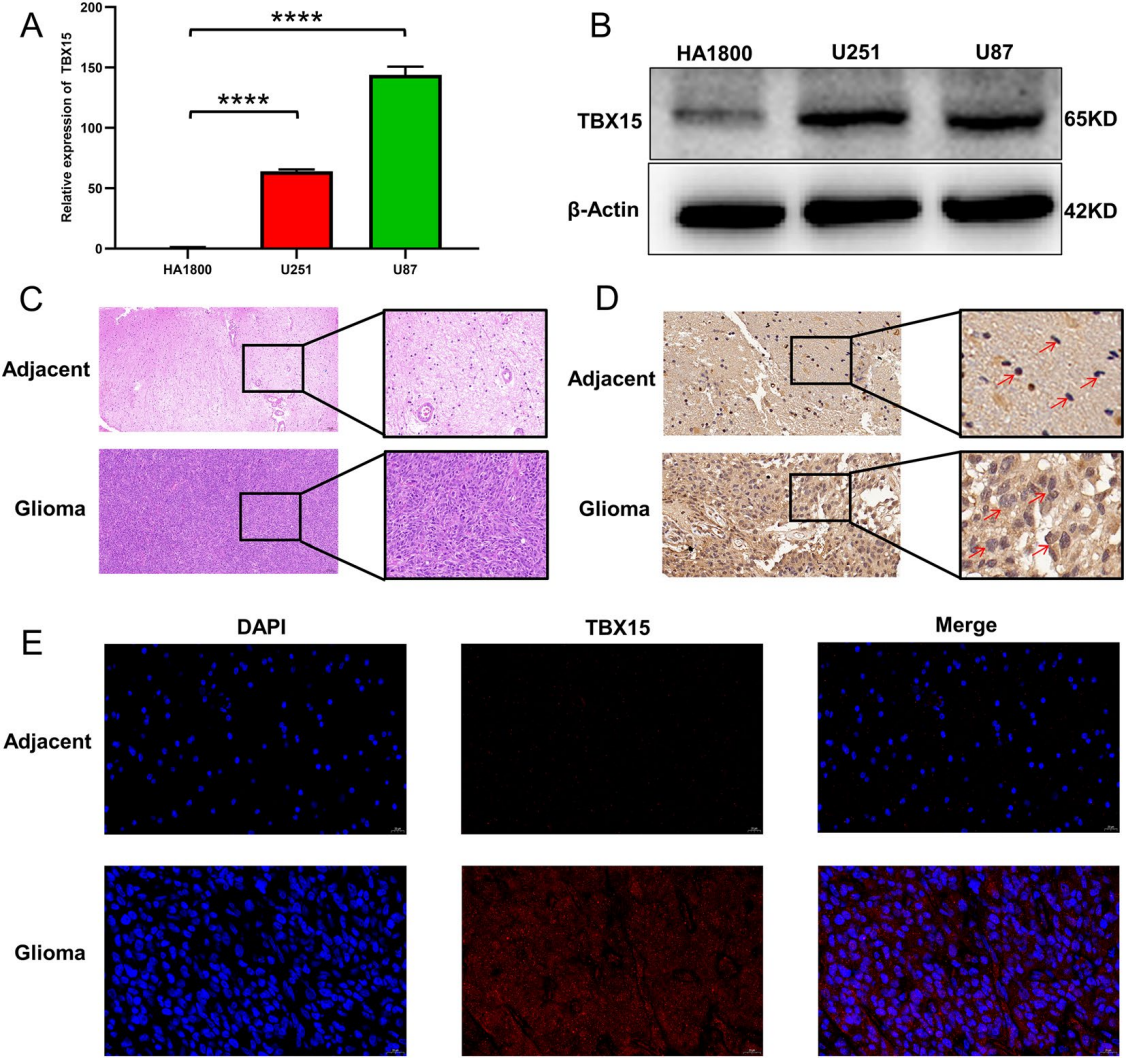
TBX15 expression is increased in glioma cells and tissues. (**A**) TBX15 expression in the relevant groups was assessed by qRT-PCR. All data are shown as the mean ± SD (three independent experiments). ****, *P* < 0.0001. (**B**) The protein expression of TBX15 in HA1800, U251, and U87 cells was normalized to β-actin by Western blot evaluation. (**C**) HE staining was used to distinguish between glioma and adjacent normal tissues. Scale bar = 100 μm. n = 3. (**D**) Representative images of TBX15 expression in adjacent glioma tissues and glioma. Scale bar = 20 μm. n = 3. (**E**) Representative images of immunofluorescence staining for TBX15 in glioma and paratumoral tissues. Scale bar = 20 μm. n = 3.

qRT-PCR analysis showed that the expression of TBX15 mRNA in glioma U87 and U251 cells was significantly up-regulated as compared with that in normal human HA1800 astrocytes (Fig. 5A). Western blot analysis showed that the expression of TBX15 protein in the two glioma cell lines was also significantly up-regulated as compared with that in HA1800 (Fig. 5B). HE staining was used to distinguish between glioma and adjacent normal tissues (Fig. 5C). TBX15 was overexpressed in glioma tissues, and undetectable or found to be only expressed at low levels in adjacent normal tissues (Fig. 5D). Immunofluorescence staining was performed in glioma and paratumoral tissues to evaluate TBX15 expression, finding that TBX15 expression was elevated in glioma tissues (Fig. 5E). Collectively, these results indicate that TBX15 was overexpressed in glioma tissues.

### TBX15 upregulation is associated with clinicopathological features of glioma

The expression of TBX15 mRNA and clinical data were obtained from the TCGA database to evaluate the correlation between TBX15 and the clinicopathologic features of glioma, including 224 WHO grade 2 cases, 245 WHO grade 3 cases, and 168 WHO grade 4 cases (Table 1). It was found that TBX15 expression was related to glioma grade (*P* < 0.001), IDH mutation status (*P* < 0.001), and age (*P* < 0.001).

**Table 1:** Clinicopathological characteristics and TBX15 expression of 695 samples from glioma patients in the TCGA database.

Characteristics	Low expression of TBX15	High expression of TBX15	*P* value
n	349	350	
WHO grade, n (%)			< 0.001
G2	152 (23.9%)	72 (11.3%)	
G3	115 (18.1%)	130 (20.4%)	
G4	40 (6.3%)	128 (20.1%)	
IDH status, n (%)			< 0.001
WT	73 (10.6%)	173 (25.1%)	
Mut	270 (39.2%)	173 (25.1%)	
1p/19q codeletion, n (%)			0.086
Non-codel	251 (36.3%)	269 (38.9%)	
Codel	96 (13.9%)	76 (11%)	
Age, n (%)			< 0.001
< = 60	300 (42.9%)	256 (36.6%)	
> 60	49 (7%)	94 (13.4%)	

We also examined the relative hazards indicated by TBX15 in terms of glioma prognosis by Cox regression analysis to see whether TBX15 was a risk factor. As shown in Table 2, high TBX15 expression was linked with a significantly increased risk of mortality in glioma patients as compared with those with low TBX15 expression by univariate Cox regression analyses (*P* < 0.001). Multivariate Cox regression analysis found that when TBX15 high expression (*P* = 0.009), age (*P* = 0.003), Grade (*P* < 0.001), and IDH mutation status (*P* < 0.001) were all taken into account, TBX15 proved to be a predictor of poor survival.

**Table 2:** Cox-regression analysis of prognostic variables in glioma patients by both univariate and multivariate analyses.

Characteristics	Total(N)	Univariate analysis		Multivariate analysis	
		Hazard ratio (95% CI)	*P* value	Hazard ratio (95% CI)	*P* value
WHO grade	636		< 0.001		
G2	223				
G3	245	2.967 (1.986–4.433)	< 0.001	1.820 (1.180–2.806)	0.007
G4	168	18.600 (12.448–27.794)	< 0.001	4.477 (2.647–7.574)	< 0.001
IDH status	688		< 0.001		
Mut	442				
WT	246	8.609 (6.602–11.226)	< 0.001	3.175 (2.129–4.735)	< 0.001
1p/19q codeletion	691		< 0.001		
Non-codel	520				
Codel	171	0.225 (0.147–0.346)	< 0.001	0.670 (0.405–1.108)	0.119
Age	698		< 0.001		
< = 60	555				
> 60	143	4.696 (3.620–6.093)	< 0.001	1.591 (1.167–2.168)	0.003
TBX15	698		< 0.001		
Low	348				
High	350	2.509 (1.948–3.231)	< 0.001	1.467 (1.101–1.956)	0.009

**Figure 6:**
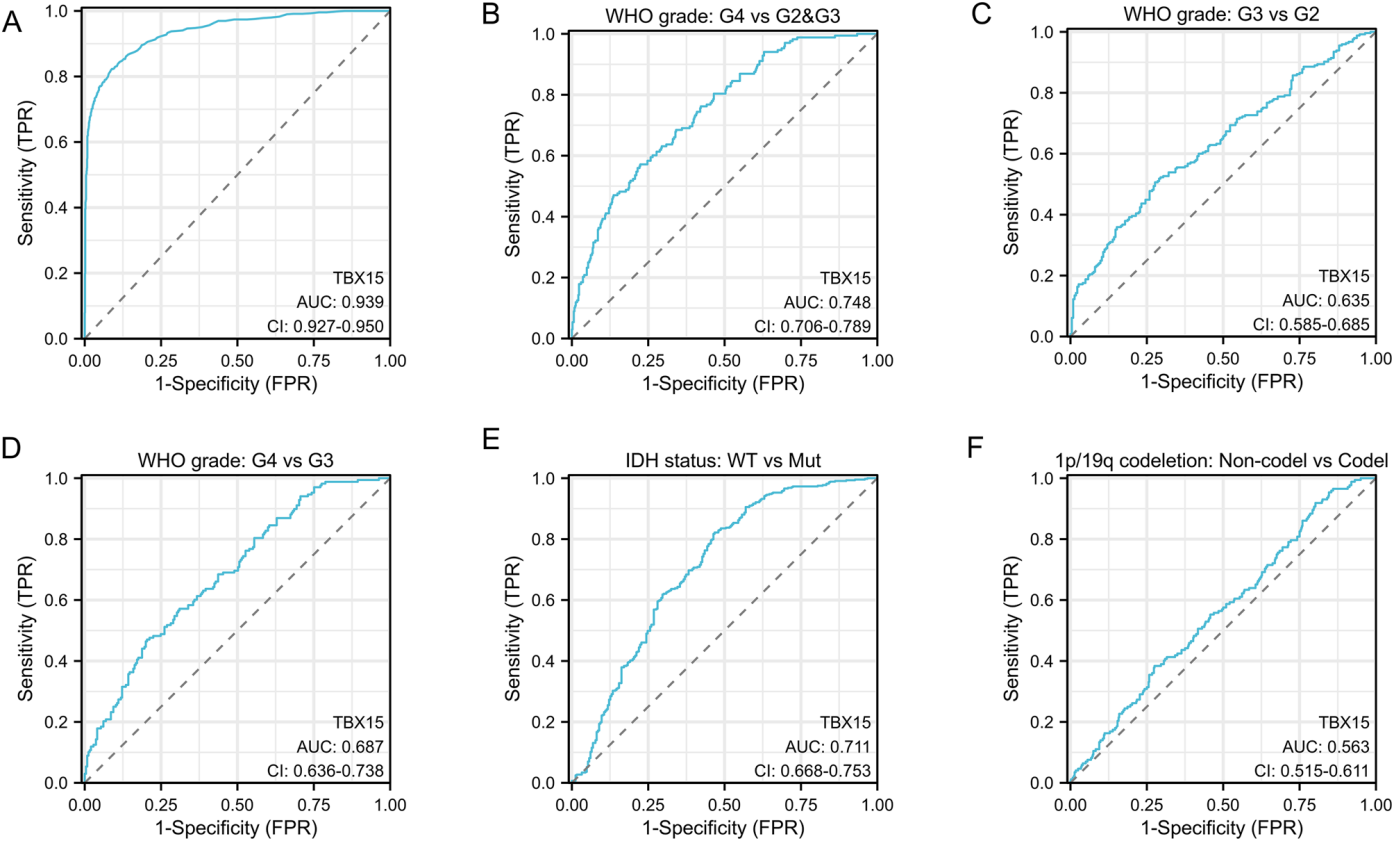
ROC curve analysis was performed to evaluate the predictive value of TBX15 for glioma diagnosis, grading, IDH status, and 1p/19q status.

To explore the diagnostic value of TBX15 in glioma, the receiver operating characteristic curve (ROC) was mapped. TBX15 exhibited high accuracy in predicting normal tissue and glioma outcomes (AUC = 0.939, CI = 0.927–0.950, Fig. 6A). TBX15 has certain accuracy in predicting the outcome of WHO level and IDH state (Fig. 6B–E). was poor in predicting 1p/19q codel and non-codel outcomes (AUC = 0.563, CI = 0.515–0.611, Fig. 6F). These results indicate that TBX15 has a certain predictive value in the diagnosis, grading, and IDH status of glioma.

### Individualized prognosis prediction models

**Figure 7:**
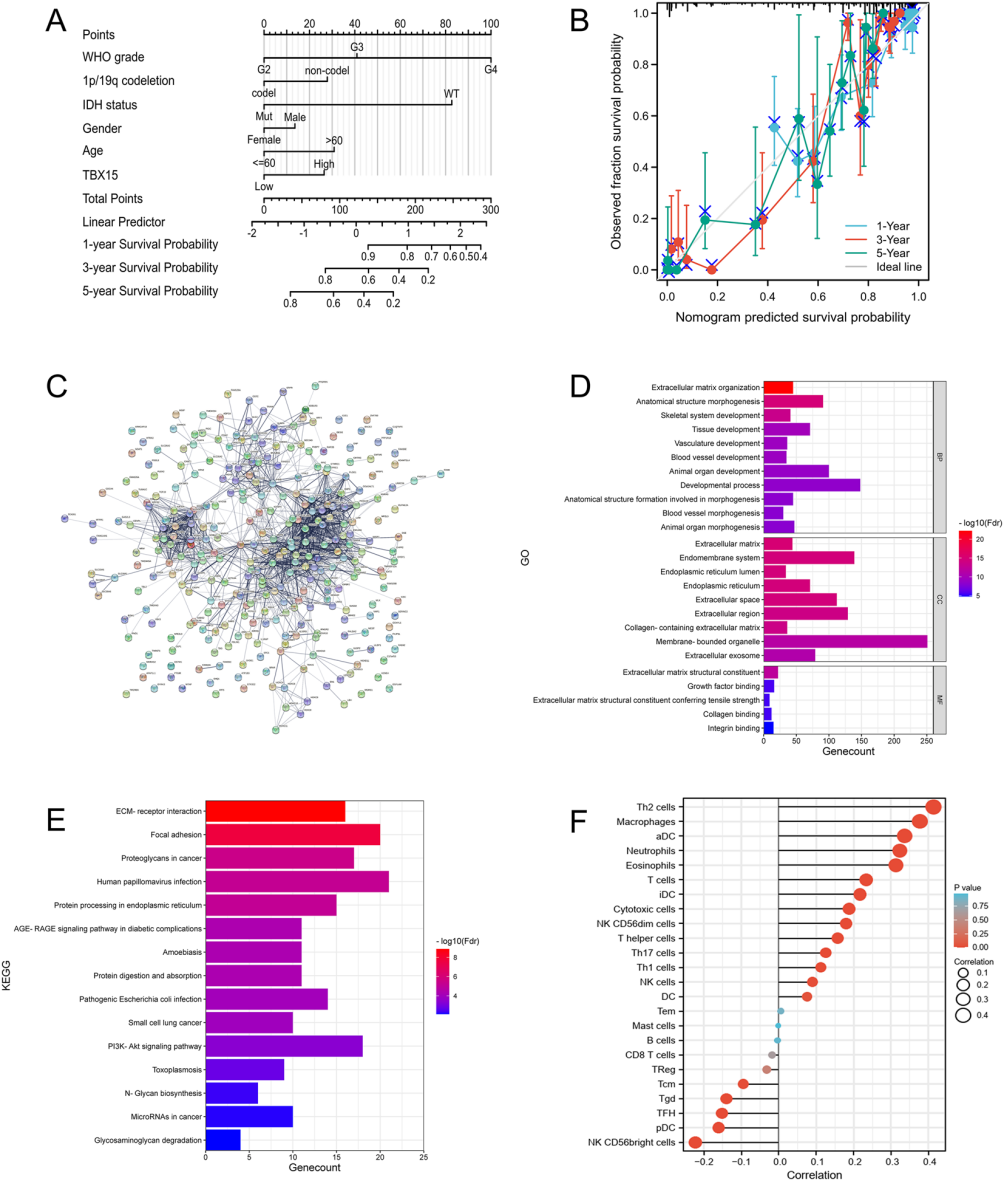
PPI network, function, and pathway enrichment analyses. (**A**) A nomogram of the glioma cohort (TCGA-GBMLGG) was used to predict overall survival. (**B**) A calibration map was used to predict 1-year, 3-year, and 5-year survival in the gliomas cohort. (**C**) TBX15 and its top 300 co-expression genes formed a network. (**D**) BP, MF, and CC enrichment studies were performed on 300 genes implicated. (**E**) Significant KEGG pathways of the top 300 genes are most positively associated with TBX15. (**F**) There was a correlation between TBX15 expression and the relative abundance of 24 immune cells. The exact Spearman's rank correlation coefficient values are shown by the size of the dots.

In many cancers, nomograms are superior to traditional TNM staging for prognostic assessment of individual patients^10^. In this study, we generated a nomogram on WHO grade, 1p/19q codeletion, IDH status, gender, age, and TBX15 to predict the probability of 1-, 3- and 5-year OS. Meanwhile, the C index was calculated as 0.851 [95% confidence interval (CI) = 0.840–0.862]. Several variables were scored based on the proportion of contribution to survival risk as shown in Fig. 7A. The calibration curve results showed that the predicted survival rate was closely related to the observed survival rate (Fig. 7B).

### Correlation and enrichment analyses

We used TCGA data to conduct correlation analysis between TBX15 and other genes in glioma and selected the top 300 genes most positively correlated with TBX15 to construct a PPI network through the STRING database (Fig. 7C). Then, we used the STRING database to predict the function of TBX15, including related pathways, based on the first 300 genes. Animal organ morphogenesis, blood vessel morphogenesis, extracellular matrix, extracellular space, endoplasmic reticulum, endoplasmic reticulum lumen, endomembrane system, and extracellular region were among the gene words associated with TBX15, according to functional enrichment and GO analyses (Fig. 7D). In addition, KEGG pathway analysis revealed an enrichment and crosstalk of the top 300 genes in ECM-receptor interaction, proteoglycans in cancer, human papillomavirus infection, and PI3K-Akt signaling pathway (Fig. 7E). Reactome pathway study revealed that extracellular matrix organization, ECM proteoglycans, collagen biosynthesis and modifying enzymes, MET promote cell motility, MET activates PTK2 signaling, Signaling by MET, and Immune System were all considerably enriched (Supplementary Fig. 1A). Wiki pathways revealed that the miRNA targets in ECM and membrane receptors, VEGFA-VEGFR2 signaling pathway, miR-509-3p alteration of YAP1/ECM axis, focal adhesion, PI3K-Akt-mTOR-signaling pathway, and PI3K-Akt signaling pathway were significantly enriched (Supplementary Fig. 1B). In addition, GSEA analysis was performed for the above representative pathways in TCGA-GBM using the CAMOIP database^11^ (Supplementary Fig. 1C–H).These results demonstrated that TBX15 was related to many malignant tumor-related pathways in glioma.

### TBX15 expression is associated with immune cell infiltration

**Figure 8:**
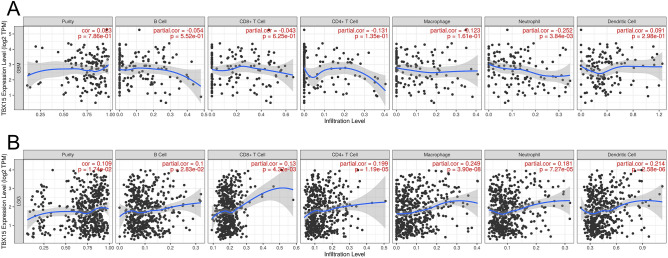
TBX15 expression is associated with immune cell infiltration. (**A**) Correlation between TBX15 expression and immune cell invasion in glioblastoma (GBM). (**B**) Correlation between TBX15 production and immune cell invasion as shown in LGG.

Tumor-infiltrating immune inflammatory cells are crucial to the development of tumors and the progression of malignancies^12^. Immune cells interact bidirectionally with tumor cells to promote multiple aspects of glioma development, including proliferation, angiogenesis, immune escape and therapeutic resistance^13^. We also used ssGSEA to investigate the connection between the TBX15 gene level and the degree of immune cell infiltration. As shown in Fig. 7F, TBX15 mRNA level was associated with immune cell infiltration, and TBX15 mRNA expression was positively correlated with Th2 cell infiltration (R = 0.414, *p* < 0.001), macrophages (R = 0.377, *p* < 0.001), aDC (R = 0.336, *p* < 0.001), neutrophils (R = 0.323, *p* < 0.001), eosinophils (R = 0.313, *p* < 0.001), and T cells (R = 0.234, *p* < 0.001). ssGSEA also demonstrated that TBX15 mRNA expression was negatively correlated with NK CD56bright cell the infiltration (R = − 0.223, *p* < 0.001), pDC (R = − 0.161, *p* < 0.001), TFH (R = − 0.151, *p* < 0.001), and TFH (R = − 0.141, *p* < 0.001), while TBX15 mRNA expression was not correlated with the infiltration of TReg (R = − 0.032, *p* = 0.394), CD8 T cells (R = − 0.018, *p* = 0.644), and B cells (R = − 0.003, *P* = 0.933), and Mast cells (R = − 0.001, *p* = 0.975). We also reviewed the TIMER database to see whether there was a link between TBX15 mRNA expression and the six categories of immune cells that infiltrated tumors. TIMER analysis revealed that TBX15 mRNA expression in GBM was negatively linked with neutrophil infiltration (R = − 0.252, *p* = 0.004) but not with the infiltration of B cells (R = − 0.054, *p* = 0.552), CD8 + T cells (R = − 0.043, *p* = 0.625), CD4 + T cells (R = − 0.131, *p* = 0.135), macrophages (R = − 0.123, *p* = 0.161), and DCs (R = 0.091, *p* = 0.298) (Fig. 8A). TBX15 mRNA expression in LGG was positively correlated with the infiltration of B cells (R = 0.1, *p* = 0.028), CD8 + T cells (R = 0.13, *p* = 0.004), CD4 + T cells (R = 0.199, *p* < 0.001), macrophages (R = 0.249, *P* < 0.001), neutrophils (R = 0.181, *p* < 0.001), and DCs (R = 0.214, *p* < 0.001) (Fig. 8B). Subsequently, we further investigated the correlation between TBX15 and immunosuppressive genes using TCGA glioma data and found that TBX15 was positively correlated with most immunosuppressive genes (Supplementary Fig. 1I). These findings suggest that a good amount of TBX15 production was linked to the immunosuppressive condition of gliomas.

## Conclusions

Increasing evidence has shown that T-box overexpression facilitates the immortal proliferation of tumor cells and induces tumor cells to avoid apoptosis, thereby accelerating the malignant transformation of malignant tumors^14–16^. The expression of TBX15 mRNA was increased in ccRCC tissues as compared with that in healthy tissues, and the survival rate of ccRCC patients with significant TBX15 expression was significantly lower than that of those with low TBX15 expression^9^. TBX15 was the downstream functional target of miR-212-5p, up-regulation of miR-212-5p reduced the expression level of TBX15, and the proliferation, cell cycle, cell invasion, and migration of ccRCC cells were significantly restricted^5^. In thyroid cancer cells, three putative NF-κB matching sites were found in a 600-bp segment of TBX15's 5'flanking region, in response to the ectopic expression of TNF-α or NF-κBp65^6^. NF-κBp65 directly interacted with the 5' distal regulatory domain of TBX15 as a regulatory element of TBX15 to boost TBX15 mRNA production in thyroid cancerous cells^7^. NF-κB promoted the anti-apoptotic activity of TBX15, suggesting that TBX15 may be a downstream anti-apoptotic gene. Interestingly, low levels of TBX15 protein are usually expressed in prostate cancer and ovarian cancer, which may be related to hypermethylation in the promoter region of TBX15^8,17^. Hypermethylation of TBX15 may provide a possible biomarker for early detection, progression, and response to therapy, and may have a major influence on reducing mortality from this disease. In addition, KIF2C was targeted by TBX15/miR-152, which inhibited autophagy and glycolysis in DOX-resistant breast cancer cells^18^.

According to our results of TCGA and GTEx data based on UCSC Xena, TBX15 is expressed differently in different tumors compared to normal tissues. Different levels of TBX15 expression in distinct tumor types may imply their different biological functions and processes. In addition, TBX15 was highly expressed in gliomas in the GSE16011. We also discovered that elevation of TBX15 expression in glioma was associated with a poor prognosis.

Tumors can become fully vascularized by inducing angiogenesis or absorbing normal tissue blood vessels, allowing them to sustain the vascular system for tumor growth, invasion, and metastasis^19^. The GO results showed that TBX15 was closely related to the developmental process of animal organs in terms of the morphogenesis, blood vessel morphogenesis, extracellular matrix, extracellular space, endoplasmic reticulum, endoplasmic reticulum lumen, endomembrane system, and extracellular region. At the same time, pathways analysis showed that TBX15 was involved in ECM-receptor interaction, proteoglycans in cancer, human papillomavirus infection, protein digestion and absorption, AGE-RAGE signaling pathway in diabetic complications, PI3K-Akt signaling pathway, extracellular matrix organization, ECM proteoglycans, collagen biosynthesis and modifying enzymes, MET promotes cell motility and activates PTK2 signaling. Furthermore, the ability of tumors to evade immune destruction is one of their most distinguishing features. Currently, the biological role of TBX15 in tumors is still very limited, and the mechanism of its immunosuppression deserves further study. It was reported that the NF-κB signaling pathway upregulates TBX15 expression in cancer cells^20^. NF-κB was characterized as a key regulator of inducible gene expression in the immune system^21^. This provides a theoretical basis for our research. The link between TBX15 and immunosuppressive gene expression suggests that TBX15 may be involved in tumor immunology regulation.

Finally, TBX15 may play a role in immune cell infiltration, which may serve as an indicator to forecast the prognosis of glioma patients.

## Materials and methods

### Data collection

TBX15 expression and clinical data of TCGA pan-cancer data and GTEx were obtained from the UCSC Xena database (https://xenabrowser.net/datapages/). Tumor tissues were retrieved from TCGA, and normal tissues were merged with normal tissues from the TCGA and GTEx databases to assess TBX15 expression. The glioma microarray data were obtained from the GEO database (https://www.ncbi.nlm.nih.gov/geo/query/acc.cgi?acc=GSE16011) and GSE16011 (platform: GPL8542)^22^.

### Gene expression profiling interactive analysis(GEPIA)

GEPIA is a browser tool using TCGA and GTEx data to deliver rapid, interactive, and customizable functionality^23^. The expression of TBX15 mRNA in normal brain tissues and glioma tissues was compared and the impact on survival and prognosis was assessed by GEPIA.

### Protein-protein interaction (PPI) network and functional enrichment analysis

The STRING database (STRING; http://string-db.org) (version 11.5) was designed to collect all known and anticipated protein interactions, both physical and functional. Enrichment analysis was conducted by STRING using well-known categorization systems like GO and KEGG, knowing that it can offer innovative classification methods based on high flux textual data and clustering algorithms of the correlation network itself^24,25^. Using data from the TCGA, Pearson’s correlation value for TBX15 and other mRNAs in glioma was calculated, based on which the top 300 genes most closely related to TBX15 were selected and a TBX15 PPI network was formed using the STRING database. Genes in this module were visualized using GO and pathway analysis.

### Immune cell infiltration

The ssGSEA method was used to analyze immune infiltration in glioma samples using the GSVA package in R (Version 1.34.0)^26^. Neutrophils, mast cells, eosinophils, macrophages, natural killer (NK) cells, CD56dim NK cells, CD56bright NK cells, dendritic cells (DCs), immature DCs (iDCs), and activated DCs were among the 24 immunocytes studied. Based on the hallmark genes of the 24 reported immunocytes^27^, the relative enrichment percentage of each immune cell was derived from the gene expression profile of each tumor sample, and Spearman correlation coefficient analysis was used to find glioma and each relationship of immune cell subgroups. The TIMER Database (https://cistrome.shinyapps.io/timer/) was also used to investigate the relationship between TBX15 expression in gliomas and six immune infiltrates (B cells, CD4 T cells, CD8 T cells, neutrophils, macrophages, and dendritic cells)^28^.

### Cell lines and culture

Glioma U251 and U87 cells were purchased from Procell Life Science & Technology Co., Ltd (Wuhan, China). In addition, HA1800 astrocytes from a healthy person (BeNa Culture Collection, Henan, China) were cultured in DMEM-H medium (WH0021A081, Procell, Wuhan, China) with 10% fetal bovine serum (FBS, A3161002C, Gibco, Carlsbad, CA, USA). U251 cells were cultured in DMEM-H medium (WH0021A081, Procell, Wuhan, China) with 10% FBS (A3161002C, Gibco, Carlsbad, CA, USA) and 1% Penicillin (100 u/mL) with streptomycin (100 g/mL). U87 cells were cultured in DMEM-H medium (WH0021A081, Procell, Wuhan, China) with 10% FBS (A3161002C, Gibco, Carlsbad, CA, USA) and 1% Penicillin (100 u/mL) with streptomycin (100 g/mL). All cells were grown in a 37 °C 5% CO_2_ incubator with saturated humidity (3111, Thermo Scientific, Rockford, IL, USA). Every 24 h, the medium was replaced, and every 72 h the cells were removed with 0.25% trypsin for passage.

### qRT-PCR

Total RNA was isolated from cells by quantitative real-time PCR (qRT-PCR) using the EZ-10 DNAawayRNA Low-Dose Extraction Kit (Sangon Biotech, Shanghai, China) according to the manufacturer's instructions, and then converted into complementary DNA (cDNA) using the Thermo Scientific RevertAid First Strand cDNA Synthesis Kit (K1622, USA). Following the conventional quantitative PCR technique, qRT-PCR of TBX15 and -Actin was conducted in a real-time PCR machine using the NovoStart ® SYBR qPCR SuperMix Plus, using β-Actin as an internal reference. The amplification primers (Shanghai General Biotechnology Co., Ltd, Shanghai, China) and the sequence information are presented in Table 3. The relative transcription level of the target gene was calculated using the 2-^ΔΔ^CT method. The mean expression was used to distinguish the TBX15 high-expression group from the low-expression group.

**Table 3:** Primer sequences.

Gene	Sequence
TBX15-F	5′-AAAGCAGGCAGGAGGATGTT-3′
TBX15-R	5′-GCACAGGGGAATCAGCATTG-3′
β-actin-F	5′-CTGGGACGACATGGAGAAAA-3′
β-actin-R	5′-AAGGAAGGCTGGAAGAGTGC-3′

F, forward primer, R, reverse primer.

### Western blot analysis

The total protein derived by lysing the cell lines on ice in RIPA lysis buffer (BL504A, Biosharp) was mixed with Phenylmethylsulfonyl fluoride (BL507A-P, Biosharp, China). The protein level of each sample was determined using the BCA kit (BL521A, Biosharp, China). Total protein (30 g) was divided using SDS-PAGE and transferred to a polyvinylidene fluoride membrane (FFP24, Beyotime, China). The membrane was blocked with QuickBlockTM Western confining liquid for 15 min, washed with TBST three times, and incubated with primary antibodies overnight at 4 °C. After three TBST washes, the membrane was treated with the corresponding secondary antibody, then washed with TBST three times, and finally detected for specific bands enhanced chemiluminescence (BL520A, Biosharp, China), using β-actin as the chemiluminescent substrate and internal reference. The images of membranes were closely cropped prior to hybridization with antibodies. Rabbit antibody against TBX15 (abs138247, 1:2000) was used as the primary antibody, rabbit antibody against IgG (BL003A, 1:5000) as the secondary antibody, and β-actin (bs-0061R, 1:5000) as the internal control. TBX15 antibody was provided by Absin (Albicin (Shanghai) Biotechnology Co., LTD, China), and β-actin antibody was provided by Bioss (Beijing Bioss Biotechnology Co. LTD, China).

### Immunohistochemistry

This study was conducted according to the Declaration of Helsinki principles. Glioma samples from histopathologically and clinically diagnosed patients in the Neurosurgery Department of the First Affiliated Hospital of Hainan Medical University were used in this study. The study protocol was approved by the Institutional Research Ethics Committee of the First Affiliated Hospital of Hainan Medical University were obtained for the use of these clinical materials for research purposes (HYLL-2022-072). Written informed consent was obtained from each patients before participating in the study.The immunohistochemical procedure is described below. Briefly, sections adhered to slides were deparaffinized with xylene, rehydrated, submerged in EDTA antigenic retrieval buffer, treated with 3% hydrogen peroxide, incubated with 1% bovine serum albumin and then with anti-TBX15 (1:100, Absin, Shanghai, abs138247) overnight at 4 °C, using normal goat serum as the negative control. After washing with PBST (PBS + 1% tween), tissue sections were incubated with the secondary antibody (Biosharp) and streptavidin horseradish peroxidase complex (Biosharp), immersed in 3.3’-diaminobenzidine, counterstained with 10% Mayer’s hematoxylin, dehydrated, and mounted.

### Immunoflorescence

Neoplastic and paratumoral tissues were collected from glioma patients, fixed with formalin, and embedded in paraffin. The paraffin-embedded and frozen sections were used for immunofluorescence staining. The primary antibody, anti-TBX15 (Absin, abs138247) was diluted (1: 100) in PBS with 1% BSA. After overnight incubation at 4 °C, the samples were washed three times with PBS and incubated with goat anti-Rabbit IgG (H + L Alexa FluorTM Plus 647, Thermo Fisher) for 2 h at room temperature. DNA was stained with DAPI (Beyotime, China), and samples were visualized with a fluorescence microscope (Olympus, Japan).

### Statistical analysis

All data were statistically analyzed using R (version 3.6.3, available at https://www.r-project.org/). The expression of TBX15 was investigated using Mann-Whitney U test (Wilcoxon rank sum test) in unpaired samples. The chi-square test was used to explore the relationship between clinicopathological features and TBX15 expression. Log-rank tests were performed using survminer and survival package to assess the effect of TBX15 on overall survival. To quantify the risk of death, we used Cox proportional hazards modeling in both univariate and multivariate studies. Gender, age, clinical stage, and treatment were all potentially confounding variables. The pROC package was used to detect the cut-off value of ORMDL2 to plot the ROC curve. The ggplot 2 package was used for visualization. *P* < 0.05 was used to evaluate the significance.

## Supplementary Information

Supplementary Information 1.

Supplementary Information 2.

## Data Availability

All the original data used in this study are freely available on the websites or links provided in this article. The datasets and code used and/or analyzed during the current study are available from the corresponding authors on reasonable request.
