# Supplementary material for: The overexpression and clinical significance of TBX15 in human gliomas
Source: Sci Rep. 2023 Jun 16;13:9771. doi: 10.1038/s41598-023-36410-y (PMC10276039; doi:10.1038/s41598-023-36410-y)
Supplement: Supplementary file 1 — Supplementary Information 1. [file 41598_2023_36410_MOESM1_ESM.pdf]

Supplementary Material

Supplementary Figure

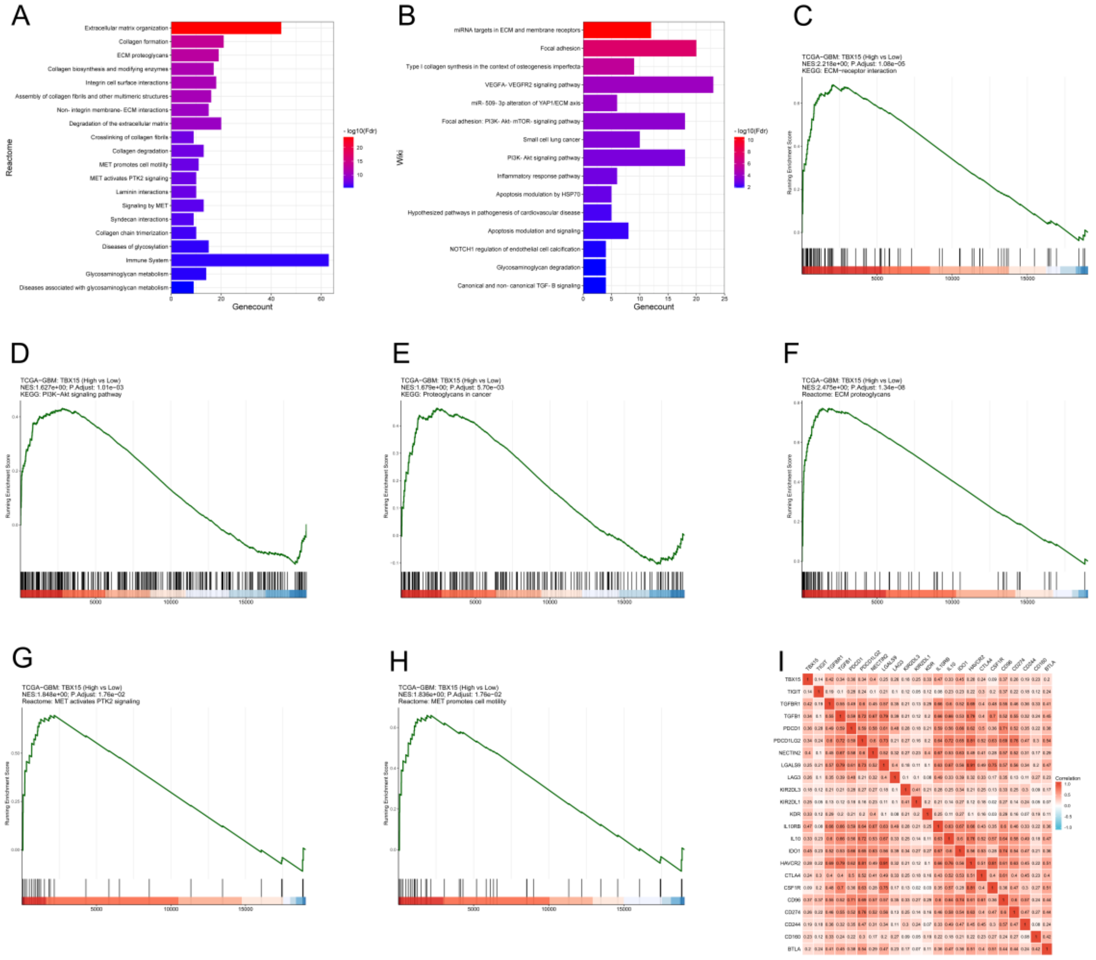

Supplementary Figure 1. (A) Significant Reactome pathways of the top 300 genes most positively associated with TBX15. **(B)** Significant Wiki pathways of the top 300 genes most positively associated with TBX15. **(C - H)** GSEA analysis of representative pathways. **(I)** TBX15was positively correlated with most immunosuppressive genes.

Supplementary table: Immunosuppressive gene

Immunosuppressive gene
TIGIT
TGFBR1
TGFB1
PDCD1
PDCD1LG2
NECTIN2
LGALS9
LAG3
KIR2DL3

KIR2DL1
KDR
IL10RB
IL10
IDO1
HAVCR2
CTLA4
CSF1R
CD96
CD274
CD244
CD160
BTLA

TBX15 was highly positively correlated with most immunosuppressive genes in glioma.

Gel images

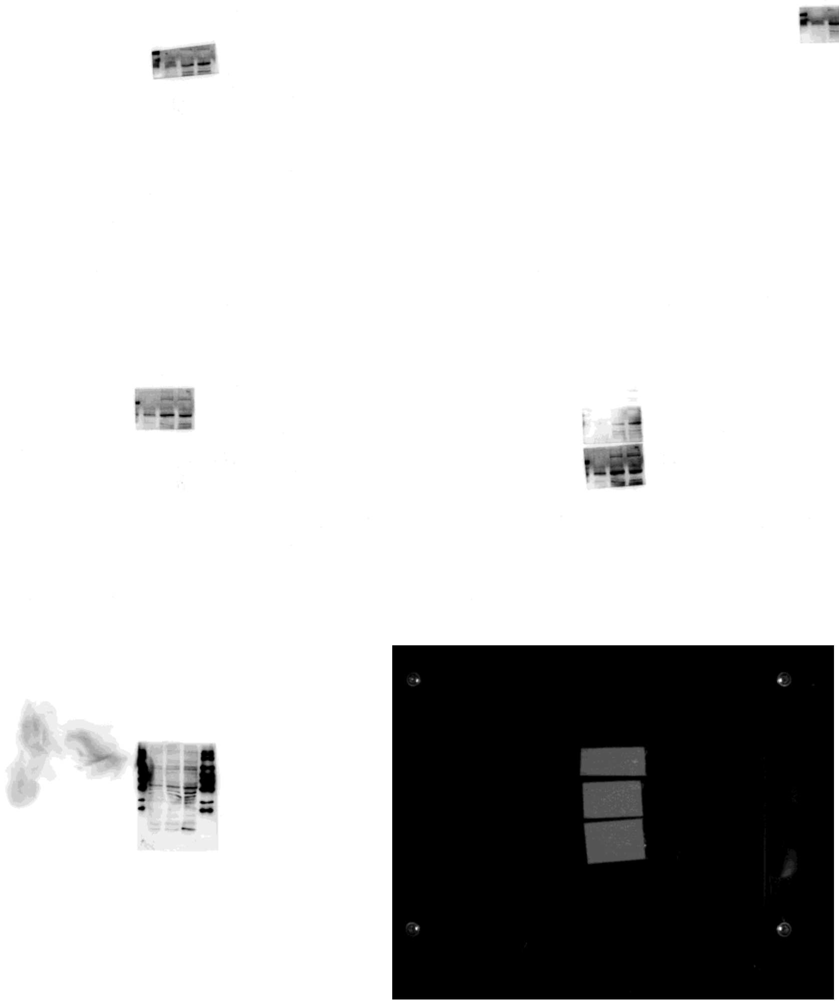

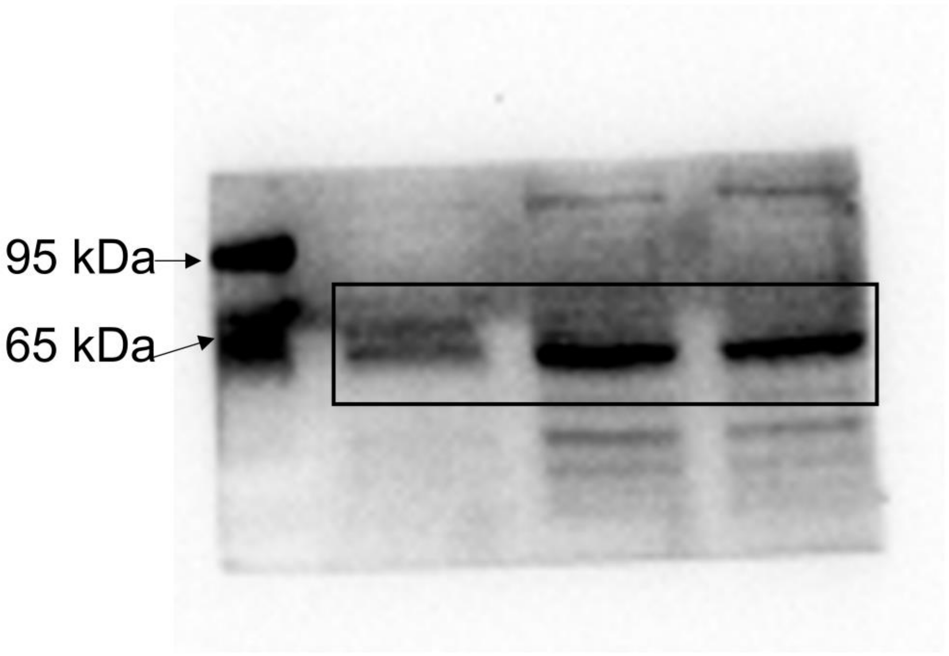

TBX15

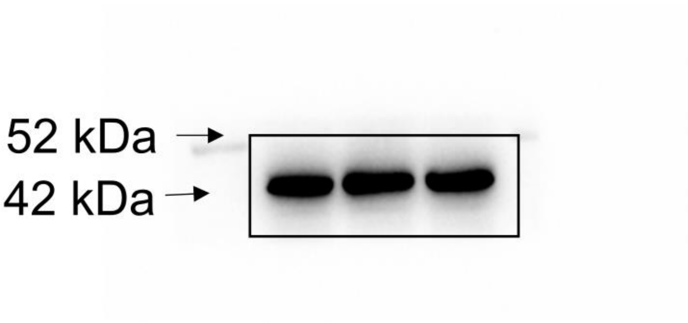

β-actin
